# Factors associated with self-rated health among migrant workers: results from a population-based cross-sectional study in Almaty, Kazakhstan

**DOI:** 10.1007/s00038-017-0944-y

**Published:** 2017-02-23

**Authors:** Pam Kumparatana, Francine Cournos, Assel Terlikbayeva, Yelena Rozental, Louisa Gilbert

**Affiliations:** 1 Mailman School of Public Health, Columbia University, New York, NY USA; 2 School of Social Work, Columbia University, New York, NY USA; 3 Global Health Research Center of Central Asia (GHRCCA), Almaty, Kazakhstan

**Keywords:** Self-rated health (SRH), Migrant workers, Central Asia, Kazakhstan

## Abstract

**Objectives:**

To determine factors associated with SRH among migrant workers in Almaty, Kazakhstan.

**Methods:**

In 2007, 805 vendors were screened. Approximately half were eligible (*n* =450), defined as at least 18 years old, a worker/owner in a randomly selected stall, having traveled 2 + hours outside of Almaty within the past year, and being an internal/external migrant. 28 non-migrants were excluded, leaving 422 participants. Logistic regression was used to examine the relationship between SRH, mental health, and psychosocial problems.

**Results:**

Approximately 46% reported having poor or fair SRH. Clinical depression (OR 0.859, 95% CI 0.342–2.154), alcohol problems (OR 1.169, 95% CI 0.527–2.593), and legal status (OR 0.995, 95% CI 0.806–1.229) were not significantly associated with SRH, nor was exposure to interpersonal violence among women (OR 1.554, 95% CI 0.703–3.435). After adjusting for key variables, only ethnicity and social support were found to be significantly protective against poor or fair SRH.

**Conclusions:**

SRH was not a comprehensive health measure for these Central Asian migrant workers. More specific questions are needed to identify mental illness and interpersonal violence.

## Introduction

Self-rated health (SRH) is a common, non-invasive way to obtain a general perspective on the health of individuals. Often assessed by the single-item measure “in general would you say your health is excellent, very good, good, fair, or poor?”, SRH is the most widely used measure of health across a range of survey research studies (Garbarski 2016). Research has shown that SRH is a strong predictor of health outcomes and mortality, independent of many biological and physical factors (Jylha 2009; Mavaddat et al. 2011; Kaplan and Baron-Epel 2003; Supiyev et al. 2014). In population studies, SRH has been shown to be a feasible, inclusive, and informative measure of health (Jylha 2009; Abikulova et al. 2013; Idler and Benyamini 1997). A review of 27 community studies showed SRH was a predictor of mortality in nearly all of the studies, even after controlling for other relevant health indicators and covariates known to predict mortality (Idler and Benyamini 1997; Surkan et al. 2009). While SRH may be influenced by age and culture, it can still be used as a valid measure of health status, and has been proposed as a global assessment (Jylha 2009; Mavaddat et al. 2011). Therefore, asking a single question about SRH is an efficient way of determining who may be at risk for poor health outcomes.

Because of its subjective nature, the specific factors that determine SRH remain uncertain. In many studies, physical health problems are more strongly associated with poor or fair SRH than mental health problems or social functioning (Mavaddat et al. 2011; Krause and Jay 1994; Shields and Shooshtari 2001; Fylkesnes and Forde 1978). Reporting poor health is related to a high number of doctor’s visits (Surkan et al. 2009). However, other studies find that poor social functioning, including lack of social support and experiencing interpersonal violence (IPV), significantly contribute to poor or fair reports of SRH, particularly for women (Surkan et al. 2009; Sundaram et al. 2004; Lown and Vega 2001). SRH has also been correlated with depression, and can be influenced by life style factors such as alcohol abuse, and psychosocial factors, including IPV and social support (Shields and Shooshtari 2001; Fylkesnes and Forde 1978; Kosloski et al. 2005; Tessler and Mechanic 1978; Manor et al. 2001). In summary, although measures of mental health and physical health have each been shown to be independent determinants of SRH, physical health measures are more consistent predictors (Tessler and Mechanic 1978; Sing-Manoux et al. 2006).

One population where obtaining SRH information may be extremely beneficial is migrant workers, a disadvantaged population in terms of health and access to health care. Migrant workers are frequently separated from their families and other social support for extended periods. They often experience job insecurity, substandard housing, poor working conditions, low wages and problems related to their undocumented status. Additional stress may result from difficulties adjusting to unfamiliar cultures (Zhong et al. 2015). A systematic review of maternal health care among migrants has shown that those without legal status have reduced access to health care and increased risks for negative physical and mental health outcomes, such as maternal death, stillbirth, early neonatal death, depression, schizophrenia, and post-traumatic stress (Almeida et al. 2013).

Worldwide, most studies report worse health access and outcomes for migrants who lack legal status. For example, migrants living on Mayotte Island, a French island in the Indian Ocean, reported barriers to healthcare related to their unstable living conditions, including their illegal residence status (Florence et al. 2010). Two studies conducted in Germany of migrants from many different backgrounds found that illegal status resulted in delays in seeking care for acute and chronic medical conditions, lower quality care, difficulties accessing a regular supply of medication for chronic illnesses and poorer physical and mental health outcomes (Castaneda 2009; Kuehne et al. 2015). However, some studies have suggested that the impact on health of residing in a country without legal status may be largely explained by socioeconomic and psychosocial factors rather than legal status itself (Pikhart et al. 2010). Given the range of findings, more empirical research is needed to achieve greater clarity about what SRH represents in the general population and among migrants.

Migrant health is of particular concern in Kazakhstan. Following the dissolution of the Soviet Union, Kazakhstan experienced high rates of unemployment, hyperinflation, and decreased life expectancy (Brainerd 2001; Surkan et al. 2009; Pikhart et al. 2010). To transform its transitioning economy, Kazakhstan adopted an aggressive strategy, drawing in large foreign investments and many migrant workers (Alam and Banerji 2000; Ismayilova et al. 2014). As a result, Kazakhstan has become the fastest growing economy in Central Asia over the past decade and is the third top destination for migrants from the Eastern European and Central Asian regions (Alam and Banerji 2000; Ismayilova et al. 2014). In 2010, Kazakhstan hosted over three million migrants, accounting for approximately 20% of the country’s population (Ismayilova et al. 2014). Approximately 54% of migrant workers in Kazakhstan are female and most come from the neighboring countries of Kyrgyzstan, Uzbekistan, and Tajikistan (Ismayilova et al. 2014; Laruelle 2008). There has also been a large influx of internal mostly rural migrants moving to Almaty, the country’s largest city (Ismayilova et al. 2014; Laruelle 2008).

Health research in migrant populations in Central Asia has primarily focused on HIV and tuberculosis; research regarding SRH in migrant populations living and working in this region is scarce (Huffman et al. 2012; El-Bassel et al. 2011). This study aims to address a critical gap in the literature by looking at SRH in a population of migrant workers in Almaty, Kazakhstan. We compared migrant workers who reported poor or fair health to migrants who reported good, very good or excellent health. We sought to determine whether socio-demographic factors, legal status, having social support, having a regular doctor, and the presence of common mental health and psychosocial factors (i.e., clinical depression, alcohol problems, and exposure to IPV) predicted worse SRH. While the literature is conflicting, we hypothesized that migrant workers who were reporting poor mental health, experiencing psychosocial problems, and/or lacked legal status would be more likely to report fair/poor health than those who were not experiencing these issues.

## Methods

### Study design and study population

Respondents were recruited from Barakholka Market, the largest market in Almaty, between July and October 2007. The market employs approximately 30,000 vendors, and participants were recruited from the five largest submarkets that contained the largest number of migrant workers. Geo-mapping was used to create a numbered list of all the stalls at these submarkets and 435 stalls were randomly selected from this list (Ismayilova et al. 2014; Gilbert et al. 2015; El-Bassel et al. 2011). Trained recruiters approached 920 vendors, with 805 vendors agreeing to participate in the screening interview (approximately 88%); approximately half (*n* =450, 52%) were eligible (Ismayilova et al. 2014; Gilbert et al. 2015; El-Bassel et al. 2011). Eligible participants were (1) at least 18 years old; (2) employed as workers or owners in randomly selected stalls; (3) people who traveled two or more hours outside of Almaty within the past year; (4) not a citizen of Kazakhstan (external migrant) or maintained a permanent residence two or more hours from Almaty (internal migrant) (Ismayilova et al. 2014); Gilbert et al. 2015; El-Bassel et al. (2011). Of those recruited, 28 were non-migrants and were excluded, leaving 422 participants for this population-based cross–sectional study. The original study protocol was approved by the Institutional Review Board of Columbia University and the Ethnics Board of the Kazakhstan School of Public Health in Almaty (Ismayilova et al. 2014; Gilbert et al. 2015; El-Bassel et al. 2011).

### Measures

Data were collected using interviewer-administered surveys, and interviews took place in the study’s private research office in the marketplace, approximately 2 weeks after participants were screened. The survey instrument was developed in English, translated into Russian, back-translated into English and piloted in Russian with 5 female and 5 male market workers. Participants were compensated 1500 Kazakhstani tenge/KZT per interview (equivalent to $10USD).

Sociodemographic data included age, ethnicity, gender, educational attainment, marital status, and legal status, the latter based on participants’ self-reported immigration status. Two questions assessed participants’ access to healthcare: did they need to see a doctor for an illness or condition in the past year, but did not; and if they had a regular doctor. Participants were asked about the number of friends, neighbors, coworkers, and the number of family members, that they could rely on for support, advice or help. Responses for both questions were collapsed into ordinal variables.

Depression was assessed by the Brief Symptom Inventory (BSI) Depression subscale (Derogatis and Melisaratos 1983; Derogatis 2001), which measured how participants felt in the past week using six items rated on a 5-point scale (e.g., *Thoughts of ending your life; Feeling hopeless about the future*). The scale has strong internal consistency (*α*=0.877). The raw score totals were converted to uniform T-scores with a mean of 50 and a standard deviation of 10 (Derogatis 2001). Based on reaching the clinical cut-off score for depression (T-score > 63), the variable was dichotomized into yes (coded as 1) or no (coded as 0). Problems with alcohol use were assessed using the well-known 4-question CAGE screening questionnaire for alcohol use disorders (Mayfield et al. 1974; Ewing 1984; O’Brien 2008). A CAGE score ≥ 2 denotes problems with alcohol (Ewing 1998).

Women were also asked questions about their lifetime experiences of physical or sexual IPV using the sexual, injurious, and physical IPV subscales of the Conflict Tactics Scales questionnaire (CTS2). Internal consistency of the CTS2 subscales ranges between 0.79 and 0.95 (Gilbert et al. 2015). For the data analysis, all the IPV variables were combined into one variable that denoted ever or never experiencing any IPV, with no, it never happened being coded as 0 (never’) and any report of IPV in the past being coded as 1 (=‘ever’).

### Outcome variable

Participants answered the question ‘How would you rate your overall quality of health?’ Response options were ‘excellent, very good, good, fair, or poor.’ This ordinal variable was recoded into a dichotomized variable, with excellent/very good/good health coded as 0 and fair/poor health coded as 1.

### Statistical analysis

All data analysis was conducted using SAS 9.3 (SAS Institute 2011).

Using both Student’s *t* test and the Chi-squared test, bivariate analyses were conducted to analyze the differences between SRH (excellent/very good/good vs. fair/poor) and participant age, sex, educational attainment, marital status, legal status, ethnicity, social support from friends, neighbors, or coworkers, and social support from family members (Table 1). For the purposes of data analysis, age was recoded into a dichotomized variable, with ‘25 and below’ being coded as 0 and ‘26 and above’ coded as 1. Participants who reported being Chinese, Korean, Turkish, Indian, Iranian, Russian, or Other were combined into the “Other” ethnicity category. Bivariate analyses were also conducted to analyze the differences between SRH and various health outcomes, including clinical depression and alcohol problems (Table 2). With female respondents, bivariate analyses were conducted on female specific health outcomes related to IPV (Table 3). All *p* values reported were for two-sided significance tests with a *p* value of <0.05 regarded as statistically significant.

**Table 1:** Socio-demographic characteristics of a random sample of migrant workers from Almaty, Kazakhstan (July–October 2007)

Variables	Poor/fair health (*n* =194)	Excellent/very good/good health (*n* =228)	Total (*n* =422)	*t* test/${\chi }^{2}$
Socio-demographic characteristics [*n*, (percent, %)]				
Age in years, mean (range, SD)	27.69 (18–36, SD =4.97)	27.88 (18–42, SD =4.71)	27.7 (SD =4.8)	0.41
Age in categories				−0.0002
25 and below	62 (31.96%)	73 (32.02%)	135 (31.99%)	
26 and above	132 (68.04%)	155 (67.98%)	287 (68.01%)	
Gender				1.91
Female	105 (54.12%)	108 (47.37%)	213 (50.47%)	
Male	89 (45.88%)	120 (52.63%)	209 (49.43%)	
Education				0.4163
Less than high school	134 (70.90%)	163 (73.76%)	297 (72.44%)	
More than high school	58 (26.24%)	55 (29.10%)	113 (27.56%)	
Marital Status				0.9915
Single, never married	53 (27.46%)	53 (23.25%)	106 (25.18%)	
Married	122 (63.21%)	152 (66.67%)	274 (66.08%)	
Separated, widowed, or divorced	18 (9.33%)	23 (10.09%)	41 (9.74%)	
Legal status				7.7375**
Legal Resident of Kazakhstan	56 (29.32%)	96 (42.48%)	152 (36.45%)	
Other	135 (70.68%)	130 (57.52%)	265 (63.55%)	
Ethnicity				56.5839***
Kazakh	56 (28.87%)	33 (14.54%)	89 (21.14%)	
Kyrgyz	44 (22.68%)	23 (10.13%)	67 (15.91%)	
Uzbek	58 (29.90%)	56 (24.67%)	114 (27.08%)	
Tajik	10 (5.15%)	13 (5.73%)	23 (5.46%)	
Karakalpak	7 (3.61%)	39 (17.18%)	46 (10.93%)	
Other	19 (9.79%)	63 (27.75%)	82 (19.48%)	
Number of friends, neighbors, coworkers for support, advice, help				18.5968**
0	55 (28.50%)	29 (12.78%)	84 (20.00%)	
1	49 (25.39%)	55 (24.23%)	104 (24.76%)	
2	46 (23.83%)	79 (34.80%)	125 (29.76%)	
≥ 3	43 (22.28%)	64 (28.19%)	107 (25.48%)	
Number of family members for support, advice, help				31.2163***
0	75 (38.86%)	39 (17.18%)	114 (27.14%)	
1	54 (27.98%)	59 (25.99%)	113 (26.90%)	
2	26 (13.47%)	47 (20.70%)	73 (17.38%)	
≥ 3	38 (19.69%)	82 (36.12%)	120 (28.57%)	

** *p* ≤ 0.05; *** *p* ≤ 0.0001

**Table 2:** Health and mental health outcomes based on self-rated health status from a random sample of migrant workers in Almaty, Kazakhstan (July–October 2007)

Variables	Poor/fair health (*n* =194)	Excellent/very good/good health (*n* =228)	Total (*n* =422)	*t* test/${\chi }^{2}$
Health and mental health outcomes [Frequency, *n* (percent, %)]				
In the past year, needed to see doctor for an illness or condition, but did not				1.3701
Yes	93 (48.19%)	96 (42.48%)	189 (45.11%)	
No	100 (51.81%)	130 (57.52%)	230 (54.89%)	
Have a regular doctor				4.3639**
Yes	5 (2.59%)	16 (7.05%)	21 (5.00%)	
No	188 (97.41%)	211 (92.95%)	399 (95.00%)	
Alcohol use disorders (CAGE)				0.4726
Alcohol problem	19 (9.79%)	18 (7.89%)	37 (8.77%)	
No alcohol problem	175 (90.21%)	210 (92.11%)	385 (91.23%)	
Depression				0.4016
Yes	14 (7.22%)	13 (5.70%)	27 (6.40%)	
No	180 (93.60%)	215 (94.30%)	395 (93.60%)	

**p  ≤ 0.05

**Table 3:** Female-specific health outcomes and self-rated health from a female subset of a random sample of migrant workers from Almaty, Kazakhstan (July–October 2007)

Variables	Poor/fair health (*n* =105)	Excellent/very good/good health (*n* =108)	Total (*n* =213)	*t* test/${\chi }^{2}$
Outcomes [*n* (percent, %)]				
Partner thrown something, twisted arm or hair, pushed, shoved, slapped, grabbed on purpose				1.4379
Yes	33 (31.43%)	26 (24.07%)	59 (27.70%)	
No	72 (68.57%)	82 (75.93%)	154 (72.30%)	
Partner kicked, slammed against a wall, beat, burned, or scalded on purpose				0.0092
Yes	19 (18.10%)	19 (17.59%)	38 (17.84%)	
No	86 (81.90%)	89 (82.41%)	175 (82.16%)	
Partner hit or punched something that could hurt, used or threatened to use				0.2101
Yes	19 (18.10%)	17 (15.74%)	36 (16.90%)	
No	86 (83.10%)	91 (84.26%)	177 (83.10%)	
Partner used force or the threat of force to have vaginal, anal, or oral sex				0.0792
Yes	15 (14.29%)	14 (12.96%)	29 (13.62%)	
No	90 (85.71%)	94 (87.04%)	184 (86.38%)	
Partner insist on vaginal, anal, or oral sex when participant did not want to				0.2023
Yes	21 (20.00%)	19 (17.59%)	40 (18.78%)	
No	84 (80.00%)	89 (82.41%)	173 (81.22%)	
Had a sprain, small cut, bruise, or physical pain that still hurt as a result of a fight with partner				0.3866
Yes	22 (20.95%)	19 (17.59%)	41 (19.25%)	
No	83 (79.05%)	89 (82.41%)	172 (80.75%)	
Broke a bone or passed out as a result of a fight with a partner				0.0034
Yes	8 (7.62%)	8 (7.41%)	16 (7.51%)	
No	97 (92.38%)	100 (92.59%)	197 (92.49%)	
Gone to a doctor or had to go to a doctor as a result of a fight with partner				0.3082
Yes	10 (9.52%)	8 (7.41%)	18 (8.45%)	
No	95 (90.48%)	100 (92.59%)	195 (91.55%)	
Ever experienced any type of IPV				1.0641
Yes	35 (33.33%)	29 (26.85%)	64 (30.05%)	
No	70 (66.67%)	79 (73.15%)	149 (69.95%)	

The relationships between SRH and poor mental health and psychosocial variables were examined using logistic regression models, with a poor or fair (=‘poor’) rating compared with good or very good or excellent (=‘good’) rating as the outcome. The logistic regression models were adjusted for sociodemographic variables. Depression and alcohol problems were chosen a priori to be tested for confounding, and for the subset of women, any lifetime experience of IPV was also tested. The same a priori covariates were tested for any interactions as well. A p value of < 0.05 was regarded as statistically significant for interaction terms while a >10% change between the adjusted OR and crude OR was used to determine presence confounding. There was no presence of confounding or significant multiplicative interactions present.

For the logistic regression models, the variable ‘marital status’ was dichotomized into currently (Married, coded as 1) or currently not (Single/Separated/Widowed/Divorced, coded as 0) married. There were three multivariable models that were estimated for both the overall population (Table 4) and the subset of women (Table 5). Model 1 in Table 4 shows the crude multivariable model for the overall population. The second model (Model 2) in Table 4 was adjusted for age, ethnicity, gender, marital status, educational attainment, and having a regular doctor. Model 3 in Table 4 was adjusted for the aforementioned variables and was also adjusted for social support variables. Model 1 in Table 5 shows the crude multivariable model for the subset of women. The second model (Model 2) in Table 5 was adjusted for age, ethnicity, marital status, educational attainment and having a regular doctor. Model 3 in Table 5 was adjusted for the aforementioned variables and also for social support variables.

**Table 4:** Factors associated with poor/fair SRH among migrant workers in Almaty Kazakhstan, July–October 2007 (results of multivariate logistic regression models)

Model	Beta	OR	OR 95% CI		*p* value	AIC
			LL	UL		
Self-rated health						
Excellent/very good/good	(Ref)	(Ref)	(Ref)	(Ref)		
Poor/fair						
Model 1^a^						586.570
Depression	0.2078	1.231	0.555	2.728	0.6087	
CAGE	0.2029	1.225	0.615	2.438	0.5634	
Legal status	0.0340	1.035	0.883	1.212	0.6734	
Model 2^b^						520.659
Depression	−0.0476	0.954	0.388	2.345	0.9175	
CAGE	0.1465	1.158	0.536	2.499	0.7089	
Legal status	0.0166	1.017	0.828	1.248	0.8745	
Model 3^c^						511.878
Depression	−0.1522	0.859	0.342	2.154	0.7456	
CAGE	0.1562	1.169	0.527	2.593	0.7008	
Legal status	−0.00464	0.995	0.806	1.229	0.9655	

*CAGE* CAGE questionnaire, *IPV* intimate partner violence, *OR* odds ratio, *LL* lower limit, *UL* upper limit, *AIC* Akaike Information Criterion

^a^Crude

^b^Adjusted for age, ethnicity, gender, educational attainment, marital status, and having a regular doctor

^c^Adjusted for age, ethnicity, gender, educational attainment, marital status, having a regular doctor, presence of friends, neighbors, coworkers for support and presence of family members for support

**Table 5:** Factors associated with poor/fair SRH amongst a subsample of female migrant workers in Almaty, Kazakhstan, July–October 2007 (results of multivariable logistic regression models)

Model	Beta	OR	OR 95% CI		*p* value	AIC
			LL	UL		
Self-rated health						
Excellent/very good/good	(Ref)	(Ref)	(Ref)	(Ref)		
Poor/fair						
Model 1^a^						302.489
Depression	−0.4749	0.622	0.233	1.660	0.3430	
CAGE	0.1151	1.122	0.387	3.253	0.8322	
IPV	0.3865	1.472	0.797	2.718	0.2171	
Legal status	0.1161	1.123	0.850	1.485	0.4148	
Model 2^b^						276.183
Depression	−0.5513	0.576	0.183	1.815	0.3464	
CAGE	0.2263	1.254	0.371	4.233	0.7154	
IPV	0.3382	1.402	0.669	2.938	0.37	
Legal status	0.0979	1.103	0.838	1.451	0.4840	
Model 3^c^						270.183
Depression	−0.5259	0.591	0.176	1.986	0.3950	
CAGE	0.1816	1.199	0.328	4.389	0.7839	
IPV	0.4410	1.554	0.703	3.435	0.2757	
Legal Status	0.0254	1.026	0.775	1.358	0.8591	

*CAGE* CAGE questionnaire, *IPV* intimate partner violence, *OR* odds ratio, *LL* lower limit, *UL* upper limit, *AIC* Akaike Information Criterion

^a^Crude

^b^Adjusted for age, ethnicity, gender, educational attainment, marital status, and having a regular doctor

^c^Adjusted for age, ethnicity, gender, educational attainment, marital status, having a regular doctor, presence of friends, neighbors, coworkers for support and presence of family members for support

## Results

### Sample characteristics

The sample characteristics of the 422 participants by SRH status are shown in detail in Table 1. The average age was about 28, almost three quarters had less than a high school education, two-thirds were married, and about two-thirds reported having a legal status of “other.” The bivariate analysis showed that legal status was significantly associated with SRH (*p* value <0.05) as was ethnicity (*p* value < 0.0001). The number of supportive friends, neighbors, and coworkers was found to be significantly associated with SRH (*p* value < 0.05) as was the number of supportive family members (*p* value < 0.0001).

### Health outcomes

As denoted in Table 1, about 54% of respondents (*n* =228) reported having excellent/very good/good health and the other 46% (*n* =194) reported poor/fair health. Table 2 shows health and mental health outcomes in detail by SRH status for the overall population. About 55% of participants said that in the past year, they needed to see a doctor but could not, and 95% did not have a regular doctor. The vast majority did not have an alcohol problem (91%) or clinical depression (94%). Less than 3.0% of participants with fair or poor health had a regular doctor, compared to 7.0% of those with good health. Of all these health outcomes, only having a regular doctor was found to be significantly associated with SRH (*p* value <0.05). Clearly, however, the vast majority of participants did not have a regular doctor.

Table 3 shows female-specific health outcomes by SRH status for all 213 women. Approximately 30% reported ever experiencing any type of IPV, 28% reported any physical IPV, 19% any sexual IPV, and 19% reported injurious IPV on the CTS2. However, none of these female-specific health outcomes were significantly associated with SRH.

### Multivariable models

Table 4 reports the betas and the odds ratios from the multivariable logistic regression models that were used to analyze the data from the overall population. In the original crude model (Model 1), neither the odds ratios nor the model was statistically significant. When the model was adjusted for age, ethnicity, gender, educational attainment, marital status, and access to a regular doctor (Model 2), the odds ratios for the variables of interest were not statistically significant. When the model was adjusted for both social support variables in addition to the previous variables (Model 3), the odds ratios for the variables of interest were not statistically significant. Evaluation of both Model 2 and Model 3 shows that clinical depression, alcohol problems, or lack of legal status did not predict worse SRH.

Table 5 reports the betas and the odds ratios from the multivariable logistic regression models that were used to analyze the female subset. In the original crude model (Model 1), neither the model nor the odds ratios were statistically significant. When the model was adjusted for age, sociodemographic factors and having a regular doctor (Model 2), and further adjusted for both social support variables (Model 3), the odds ratios for the variables of interest were not statistically significant. Evaluation of both Model 2 and Model 3 shows that clinical depression, alcohol problems, IPV, and legal status did not predict worse SRH among female migrant workers.

The bivariate analyses initially showed that legal status, ethnicity, having a regular doctor, social support from family, and social support from friends and others, were significantly associated with SRH. However, Model 2 (Table 4) showed that after adjusting for clinical depression, alcohol problems, legal status, age, sociodemographic factors, and having a regular doctor, only ethnicity (*p* value < 0.0001) was significantly associated with poor/fair SRH. Specifically, when compared to Kazakhs (a group of Turkic people who primarily live in Kazakhstan), Karakalpaks (a group of Turkic people who primarily live in Uzbekistan) (OR 0.15; 95% CI 0.08, 0.31) were less likely to report poor/fair SRH. Model 3 (Table 4) showed that both ethnicity (*p* value < 0.0001) and family support (*p* value =0.0078) were associated with a reduced likelihood of reporting poor/fair SRH. Again, when compared to Kazakhs, Karakalpaks (OR 0.16; 95% CI 0.08, 0.34) were less likely to report poor/fair SRH. Additionally, when compared to those who reported having no family support, those with family support were less likely to report poor/fair SRH.

For women, Model 2 (Table 5) showed that only ethnicity (p-value =0.0002) remained significantly associated with SRH. Specifically, when compared to Kazakh women, Karakalpak women (OR 0.15; 95% CI 0.06, 0.38) were less likely to report poor/fair SRH. Also for the subset of women, Model 3 (Table 5) showed that only ethnicity and family support were significantly associated with SRH. When compared to Kazakh women, Karakalpak women (OR 0.18; 95% CI 0.07, 0.48) were less likely to report poor/fair SRH. Regardless of ethnicity, women who had family support were less likely to report poor/fair SRH than those who did not.

## Discussion

In this relatively young population of migrants, only Karakalpak ethnicity and family support were predictors of good SRH when examining other key factors. Working conditions may be a factor in our ethnicity finding. Karakalpak migrants mostly sold fruits and constantly moved throughout the market, compared to other ethnic groups, who primarily sold cloth and shoes and remained stationary in the cold. It is well established that physical activity, including walking, is associated with better health (Bauman 2004; Brown et al. 2007). Furthermore, movement may have lessened the impact of the harsh weather conditions.

In contrast to our expectations, legal status, clinical depression, alcohol problems, and, for women, a history of interpersonal violence, did not make a contribution to SRH. Because clinical depression has many physical symptoms, we had imagined people suffering from this condition would report poorer health (Kosloski et al. 2005; Tessler and Mechanic 1978; Manor et al. 2001; Sing-Manoux et al. 2006). Regarding alcohol, even in young people who have not reached the point of experiencing liver damage, excessive alcohol use can nonetheless lead to a variety of more immediate medical problems, such as gastritis and accidental injury. Certainly, IPV is another source of physical injury and previous research has shown that IPV is associated with poorer SRH (Surkan et al. 2009; Sundaram et al. 2004; Lown and Vega 2001). Although it seems perplexing that none of these conditions are associated with SRH in this study, these findings are consistent with studies suggesting SRH is more likely to reflect physical functioning rather than mental health problems and psychosocial adversities (Mavaddat et al. 2011; Krause and Jay 1994; Shields 2001; Fylkesnes and Forde 1978). The positive psychosocial factor that did hold up in our study, the presence of family social support, is consistent with those studies that find social support is associated with better SRH (Surkan et al. 2009; Krause and Jay 1994; Shields 2001; Fylkesnes and Forde 1978; Lown and Vega 2001; Kosloski et al. 2005; Tessler and Mechanic 1978; Manor et al. 2001).

While having a regular doctor was a predictor of better SRH in the bivariate analyses, 95% of participants did not have one, suggesting that for this population, having regular access to healthcare may not impact SRH. We previously reported that high mobility was associated with poor utilization of health care services in this population (Ismayilova et al. 2014). Internal migrants may be as much at risk as external migrants because in Kazakhstan health care access is linked to the specific location of legal residency. This may help to explain why people of Kazakh ethnicity did not show any advantage in SRH.

It is concerning that almost half of participants in this relatively young group of migrants reported poor or fair health. Since this was not explained by the mental health problems we studied nor by IPV, the reasons for this require further study to more accurately assess their health risks. In a study among 1199 randomly selected residents of Almaty aged 45 years or older, women and those with less education had poorer SRH; however, our migrant population did not show any differences in SRH by gender or education, suggesting that they have a different profile than the general and primarily Kazakh and ethnic Russian aging population of Almaty (Abikulova et al. 2013).

Research has suggested that because of different contextual frameworks of evaluation, positive SRH is no guarantee of physical health, but poor SRH warrants further attention, and many empirical analyses indicate that good SRH and less than good ratings of SRH differ more fundamentally than just in terms of the number of specific underlying health problems (Jylha 2009; Kaplan and Baron 2003; Idler 1997; Surkan et al. 2009). Clearly, we need a better understanding of how social and structural contexts influence SRH.

### Limitations

Since all data were cross-sectional, temporality and causality cannot be established. Although we used a random sampling approach, our data may not be representative of all migrant workers employed at the market. Since certain variables were not captured (such as income, trauma, stress, food insecurity), there may be unknown confounding or mediating factors. This sample was relatively young with low percentages reporting negative mental health and psychosocial outcomes. Because IPV is a sensitive topic, female respondents may have underreported their experiences. Although our survey instrument had been translated into Russian and piloted with migrant workers, conducting all interviews in Russian may have resulted in a lack of understanding of some survey questions, and underlying cultural differences could have influenced participants’ responses.

### Conclusion

While reports of poor SRH are worrisome, reports of good SRH likely underestimate certain health problems, such as mental illness and IPV, and therefore SRH was not a truly comprehensive measure of health for this population of migrant workers. Rather, specific questions designed to detect mental illness and IPV are needed. Our migrant population had risks for poor health outcomes at relatively young ages, and the reasons for this require further study. Future studies should also examine how specific tasks performed by migrant workers affect health, as well as the relationship between SRH and the role of social networks in utilizing health and mental health services. Given that family support was protective against poor/fair SRH, future interventions incorporating social network strategies might improve the health of migrant workers and increase their utilization of healthcare services.
